# Expected value of implementation of icosapent ethyl in Sweden

**DOI:** 10.1186/s12913-025-13470-6

**Published:** 2025-09-15

**Authors:** Michaela Eklund, Lars-Åke Levin, Lars Bernfort

**Affiliations:** Unit of Healthcare Analysis, Department of Medical and Health Sciences, Linköping University, Linköping, Sweden

**Keywords:** Icosapent ethyl, Triglycerides, Cardiovascular disease, Value of implementation, Prevention, Perfect implementation

## Abstract

**Aim:**

Cardiovascular disease poses a significant health and economic challenge in Europe, yet the adoption of novel cost-effective treatments like icosapent ethyl remains limited. This study aims to estimate the absolute and relative value of eliminating suboptimal implementation of icosapent ethyl in Sweden.

**Methods:**

This study estimates the effects of optimal and actual implementation of icosapent ethyl in Sweden with a five-year perspective by combining available cost-effectiveness evidence with epidemiology data from the Swedeheart registry and Swedish health authorities. The implementation analysis is based on the expected value of perfect implementation framework which estimates the value of increasing implementation from the current level of prescriptions up to an optimal level, where 80% of patients with indication for icosapent ethyl receives it.

**Findings and interpretation:**

The results provide an upper limit of the value that could be gained by improving implementation of icosapent ethyl in five years in Sweden. Optimal implementation of icosapent ethyl in Sweden over five years could prevent 1,228 cardiovascular events, including 99 deaths, while generating a health gain of 3,538 QALYs and net value of implementation of 497 QALYs. Regional differences and delays in implementation play a major role in the suboptimal uptake of care. Enhancing implementation strategies could lead to additional gains in QALYs and significantly reduce CV events and healthcare costs.

**Conclusion:**

There is significant health, QALY and monetary gains in ensuring improved implementation of icosapent ethyl in Sweden. There is a need for tailored interventions to address factors influencing prescribing practices. However, challenges remain with a lack of clear national or local guidelines. By decreasing the large regional variations, one could enhance patient outcomes.

**Supplementary Information:**

The online version contains supplementary material available at 10.1186/s12913-025-13470-6.

## Introduction

Cardiovascular (CV) disease is a major contributor to morbidity and imposes a significant economic burden across Europe [1]. The introduction and implementation of novel CV medications in the region follow a well-structured process that may include assessment of cost-effectiveness, promotion of clinical awareness and regional policies. Despite or because of these measures and processes, real-world uptake of novel CV medications often remains delayed, leaving a considerable proportion of patients at persistently high risk for cardiovascular events [2, 3]. This suboptimal implementation, as seen with therapies such as icosapent ethyl, is frequently driven by factors including unclear clinical guidelines, limited practitioner awareness, and organizational barriers [4]. These common challenges within cardiovascular prevention underscore the urgent need for targeted implementation strategies to ensure that evidence-based treatments reach eligible patients. Icosapent ethyl reduces the risk of CV disease recurrence in statin-treated patients with elevated levels of triglycerides and/or patients with diabetes and an additional CV risk factor as observed in the Reduction of Cardiovascular Events with Icosapent Ethyl-Intervention Trial (REDUCE-IT) [5]. In REDUCE-IT, treatment with icosapent ethyl resulted in a significant reduction in major adverse cardiovascular events, including cardiovascular death, myocardial infarction, stroke, and coronary revascularization, compared to placebo. The benefits were observed despite background statin therapy, suggesting an added protective effect beyond its lipid lowering purpose. The study population shared several key clinical characteristics with typical secondary prevention patients, which supports, but does not guarantee, the generalizability of the results to real-world populations. These outcomes provide a strong rationale for considering icosapent ethyl in routine clinical practice for high-risk patients. To date, icosapent ethyl has been reimbursed and made available in five European countries: Sweden, the United Kingdom, Finland, the Netherlands, and Spain. However, within the first year following reimbursement, all these countries reported an uptake of 1% or less of their estimated reimbursed patient populations, which is both prevalent and incident patients with indication (see Supplementary table 1). Icosapent ethyl was approved by the European Medicines Agency (EMA) on 28 January 2021 and the Swedish Dental and Pharmaceutical Benefits board (Tandvårds- och läkemedelsverket [TLV]) approved and deemed icosapent ethyl to be cost-effective on the 24^th^ of March 2022 for patients diagnosed with CV disease, with statin treatment and elevated levels of triglycerides (≥1 · 7 mmol/l) [6, 7]. The Swedish cost-effectiveness analysis showed a Quality-Adjusted Life Year (QALY) gain of 0 · 27 per patient with icosapent ethyl treatment, suggesting a substantial potential benefit when compared to standard care. However, despite this demonstrated efficacy, there are yet no national guidelines or recommendations regarding icosapent ethyl in Sweden for treating patients with established CV disease and elevated levels of triglycerides. This creates a gap in translating cost-effective evidence-based treatment into routine clinical practice.

It lies in the interest and obligation of a publicly financed healthcare system to utilize cost-effective treatments to generate maximum health in realm of available (limited) resources [8]. Reimbursement decisions based on incremental cost-effectiveness ratios (ICER) are common, but these decisions do not necessarily imply that efforts are put into promoting adherence to the decision and/or clinical guidelines. The health gains and advantages of new drugs are only realized when the reimbursed drug is used in clinical practice [9–12]. Decision makers must not only make reimbursement decisions for cost-effective technologies, but also estimate the value and cost of further research and implementation strategies. A method to investigate this is the Bayesian value-of-information framework, which relates to decisions regarding the potential advantages and costs of further research and implementation strategies. Optimal implementation is reached only when the total eligible population has access to the drug, everything else is considered suboptimal or imperfect implementation. Suboptimal implementation within and across countries and regions could result from knowledge gaps, systemic barriers, attitudes, or even chance [13].

As imperfect implementation has been identified as a key challenge for healthcare systems, this study places an upper limit on the expected value of implementation strategies for icosapent ethyl in Sweden [8, 9, 13]. Those upper limits can be used by policymakers to inform investment decisions in implementation strategies by considering the cost of the implementation strategy to decide whether it is worthwhile. Clinical practice variations and uneven implementation of effective and cost-effective medical technologies are key challenges for healthcare systems that may lead to suboptimal treatment and in turn efficiency and health losses and increased costs [8, 11]. This study aims to estimate the absolute and relative value of optimal implementation of icosapent ethyl compared to standard practice in Sweden and analyse regional differences in implementation. Gains and losses with optimal and current clinical implementation are evaluated for each region in Sweden in a five-year perspective, by combining the cost-effectiveness evidence with epidemiology from the Swedeheart registry (RIKS-HIA and SEPHIA). The results present the potential impact of improved access to icosapent ethyl, underscoring the importance of strategic implementation to mitigate CV risks, save costs and enhance patient outcomes.

## Method

The Consolidated Health Economic Evaluation Reporting Standards 2022 (CHEERS 2022) guidelines were followed in preparing this manuscript, as they are widely applied in health economic research. [14].

### Expected value of perfect implementation

The implementation analysis is based on a framework developed by Fenwick et al. to estimate the expected value of perfect implementation [12]. We will estimate the value of increasing implementation from the current level of implementation up to a perfect level of implementation over a five-year period. A five-year time horizon was chosen, as this allows for capturing meaningful changes while avoiding the high uncertainty associated with longer-term projections. Shorter horizons may miss important effects, whereas beyond five years it becomes increasingly difficult to predict future developments. The level of implementation is the proportion of patients receiving icosapent ethyl. Hence, the perfect level of implementation of a cost-effective drug is 100% and of a non-cost-effective 0%. The current level of implementation is the proportion of patients currently receiving icosapent ethyl. The expected value of perfect implementation is estimated by using the incremental net benefit (INB) framework [15].

Johannesen et al. have further developed the framework to analyse specific suboptimal (imperfect) implementation strategies as well as analyse regional variations with the expected value of specific implementation strategies [11]. Expected value of specific implementation strategies estimates the value of reaching an improved level of implementation. This framework could also be applied to analyse regional differences by estimating the value of eliminating implementation differences between regions.

### Net benefits of icosapent ethyl

In comparison to the total cost or QALY gain, which presents the additional cost or health benefit associated with an intervention in comparison to standard of care, INB estimates the value of an intervention in relation to a willingness to pay (WTP) threshold. INB can be estimated in both monetary, incremental net monetary benefit (INMB), and health terms, incremental net health benefit (INHB). A technology is cost-effective at a specific WTP if the incremental net benefit is greater than 0.

We used existing cost-effectiveness evidence of icosapent ethyl to estimate the incremental net benefits (Table 1). A Swedish health economic analysis by TLV comparing icosapent ethyl as an addition to standard treatment to standard treatment with no addition was publicly available [7]. In the absence of national guidelines for the treatment of patients with CV disease and elevated triglycerides, the standard pharmaceutical treatment in the analysis is statins as monotherapy, or in combination with other blood lipid-lowering treatments.

**Table 1:** Incremental net benefits of icosapent ethyl at 6% reduction of the relative risk

	Icosapent ethyl	Standard treatment	Difference
Life years	11 · 71	11 · 41	0 · 30
QALYs	8 · 52	8 · 25	0 · 27
Drug costs SEK (€)	142,866 (12,344)	3,548 (307)	139,318 (12,037)
Other healthcare costs SEK (€)	105,141 (9,084)	129,431 (11,183)	−24,290 (−2,099)
Costs of side effects SEK (€)	5,604 (484)	4,592 (397)	1,012 (87)
Total cost SEK (€)	253,611 (21,933)	137,571 (11,898)	116,040 (10,036)
ICER			430,286
INHB (QALYs)			0.04
INMB (SEK (€))			18,960 (1,640)
Net benefits at 3 and 0% reduction of relative risk			
Reduction in RR	3%	0%	
	Diff	Diff	
QALY	0 · 33	0 · 38	
Total cost	113,087	110,134	
INHB (QALY s)	0 · 10	0 · 16	
INMB (SEK (€))	49,413	79,866	

Source [7]

The health economic model is based on a Markov-model from a healthcare perspective. The model assumed an average age of 64 years at baseline, and a lifelong time-horizon and used a standard discount rate of 3% for both costs and effects [7]. Event rates were based on results from REDUCE-IT [5], and were used to inform the number of patients with first, second, and third (or more) CV events. The primary outcome of the trial was time to a 5-point MACE event which is a composite endpoint of CV death, non-fatal MI, non-fatal stroke, revascularization, and unstable angina. Results from REDUCE-IT showed that in the secondary prevention group, that is patients with established cardiovascular disease, the primary outcome occurred for 19 · 3% of the patients in the treatment arm and 25 · 5% in the placebo arm, resulting in a hazard ratio of 0 · 73 and a relative risk reduction of icosapent ethyl of 27% compared to standard of treatment. Age- and gender-specific estimates of mortality risk due to other reasons than CV death was based on Statistics Sweden’s (SCB) life tables [16]. Age-adjusted utility weights were applied from Ara et al. [17]. The economic model did not explicitly account for waning treatment effects over time, consistent with the sustained efficacy observed throughout the REDUCE-IT trial. Discontinuation was handled through an intention-to-treat approach in REDUCE-IT and was not separately modeled in the cost-effectiveness analysis.

EMA stated that there could be a potential negative effect of pills containing mineral oil in the placebo arm, as changes in biomarkers were seen. This could also be explained by natural progression of disease or regression to the mean effects. Irrespective of a negative effect it would be limited, and the clinically meaningful effect would remain. The National Institute for Health and Care Excellence (NICE) concluded that a potential negative effect may have affected the results with 1 · 5–3% [18]. EMA and U.S. Food and Drug Administration (FDA) estimated a potential negative effect of 3% [19, 20]. Following the most conservative estimate from TLV [7], we reduced the number of CV events in the placebo by 6%, which gave a relative risk reduction with icosapent ethyl compared to the standard treatment of 20%. A willingness to pay (WTP) threshold of SEK 500,000 (€43,000) per quality-adjusted life year (QALY) was applied to estimate the INB.

### Eligible population

Although most of the eligible population is found in the primary care setting, regional clinical recommendations include initiation of treatment only through hospital specialists in the initial phase [21]. Therefore, this analysis focuses on the systematic introduction of icosapent ethyl through hospital specialist care units. To estimate the level of implementation and define the eligible population, we used epidemiology data from the Register of Information and Knowledge about Swedish Heart Intensive Care Admissions (RIKS-HIA) and Secondary Prevention after Heart Intensive Care Admissions (SEPHIA) [22, 23]. RIKS-HIA enrolls patients admitted to cardiology care units because of symptoms suggestive of acute coronary syndromes. SEPHIA enrolls patients in secondary care after Myocardial Infarction (MI). RIKS-HIA distinguishes between patients with ongoing statin treatment at hospitalization and those with statin treatment at discharge, we consider the latter in this study. The SEPHIA register consists of two follow-ups (FU), one 6–10 weeks post discharge from acute coronary care unit (FU1) and a second visit a year post discharge (FU2). We adjusted the registry data to account for their respective coverages [24]. Notably, patients older than 80 years registered in RIKS-HIA are not followed in SEPHIA. To compensate for this data gap, we applied the age distribution from RIKS-HIA to the missing SEPHIA follow-up population.

In Sweden, icosapent ethyl is indicated and reimbursed to patients with established CV disease and elevated levels of triglycerides who are on stable statin treatment. Therefore, our base-case assumes prescription at SEPHIA FU1. However, not all patients meeting indication criteria at FU1 are expected to be eligible for treatment due to contraindications such as frailty, severe heart failure, fish allergy, hypersensitivity, or severe illness. Taking these factors into account, we estimate that approximately 80% of patients with an indication at SEPHIA FU1 are eligible for icosapent ethyl treatment at the optimal implementation level. Thus, 80% of patients with indication at SEPHIA FU1 reflects the total eligible population at optimal implementation in this study. Scenario analyses were performed considering the eligible population in RIKS-HIA and SEPHIA FU2. Table 2 presents the annual number of patients at the optimal level of implementation in Sweden.

**Table 2:** Annual number of eligible patients at optimal level of implementation in Sweden and divided by region

	SEPHIA		RIKS-HIA At discharge
	Follow-up 1 (Basecase)	Follow-up 2	
Sweden	2,794	2,421	4,450
Stockholm	403	383	675
Uppsala	82	80	125
Sörmland	95	62	171
Östergötland	131	108	200
Jönköping	111	88	166
Kronoberg	75	51	88
Kalmar	70	64	132
Gotland	22	15	32
Blekinge	59	38	156
Skåne	378	323	570
Halland	77	70	138
Västra Götaland	472	409	589
Värmland*	84	75	178
Örebro	88	76	182
Västmanland	78	76	101
Dalarna	108	91	217
Gävleborg	101	93	122
Västernorrland	117	107	119
Jämtland	56	49	98
Västerbotten	77	63	145
Norrbotten	108	101	246

Note: *Värmland do not measure TG-levels

**Fig. 1:**
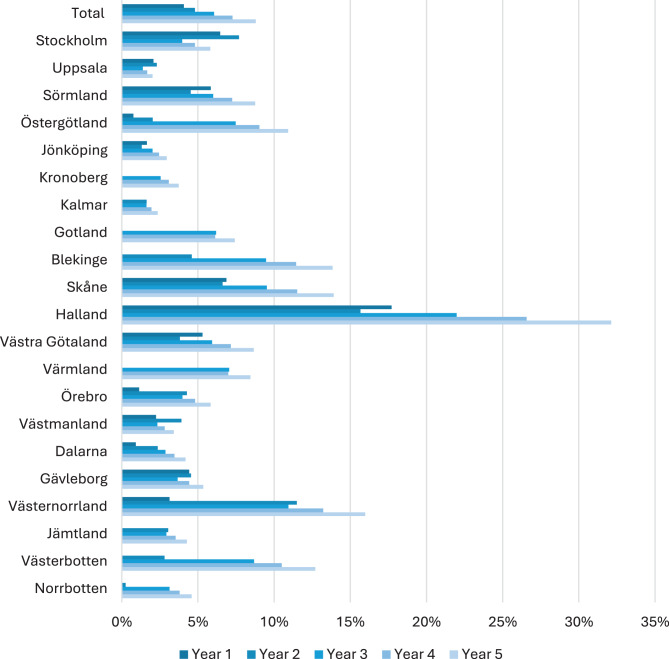
Current level of implementation in Sweden and by region based on the number of patients with indication for icosapent ethyl at the first follow-up visit post MI

Registry data from the Swedish eHealth Agency (eHälsomyndigheten) and the National Board of Health and Welfare (Socialstyrelsen) provided the number of patients who received icosapent ethyl in Sweden and in each region during the first two and a half years after the reimbursement decision. To estimate the expected level of implementation over the upcoming two and a half years, we extrapolated by applying the average annual rate of implementation observed during the initial two and a half years following the reimbursement decision, assuming this exact yearly rate continues unchanged for the subsequent two and a half years. This approach is reasonable as no major changes were observed during the initial 2 · 5 years, and significant shifts are unlikely over such a short timeframe. The data revealed that some regions did not initiate prescribing at all during the first 30 months (namely Gotland and Värmland), which is referred to as delayed implementation. To estimate the current level of implementation in these regions it was assumed that they will start implementation year 3 at the same level as the region with the lowest level of implementation; Kalmar with an average level of implementation at FU1 of 1 · 51%. The current level of implementation per year is presented in Fig. 1.

In summary, the net benefit of icosapent ethyl treatment has been presented and both the current and optimal eligible populations have been estimated. Using the EVPIM framework, we assess the potential gains from improving implementation levels over a five-year period, including addressing regional variations.

## Results

### Expected value of optimal implementation in Sweden

By reaching optimal implementation an additional 13,105 patients would be treated over five years. It is estimated that at least 1,228 CV events may be avoided in five years, including 99 deaths, 81 strokes, and 315 MIs. The total health gain of optimal implementation is estimated to be 3,538 QALYs and the total cost is 1 · 5 billion SEK, which corresponds to a QALY gain of 0 · 27 per patient and a cost of 114,460 SEK per patient The total cost and health gain results in a total net value of implementation in a lifetime perspective (Table 3) is 497 QALYs or SEK 249 million (€ 21 million), equal to a value of SEK 18,960 (€ 1,636) per patient. By improving implementation to reach 50% of the eligible population would result in 232 additional QALYs and to ensure 75% implementation would equal an additional 364 QALYs.

**Table 3:** Expected value of implementation in basecase analysis

Year	0	1	2	3	4	Total
Optimal population	2,794	2,794	2,794	2,794	2,794	13,971
Current population	114	134	169	203	246	866
Difference	2,680	2,660	2,625	2,591	2,549	13,105
Value of implementation (QALYs)	102	101	100	98	97	497
Value of implementation SEK (€) (millions)	50 · 8 (4 · 4)	50 · 4 (4 · 4)	49 · 8 (4 · 3)	49 · 1 (4 · 2)	48 · 3 (4 · 2)	248 · 5 (21 · 4)
Total health gain, QALYs	724	718	709	700	688	3,538
Improved implementation (QALYs)						
50% implementation	49	48	47	45	44	232
75% implementation	75	74	73	72	70	364
Regional differences (QALYs)						
1^st^ (Halland)	14	12	17	20	25	88
2^nd^ (Västernorrland)	−1	7	5	6	8	25
3^rd^ (Skåne)	3	2	4	5	5	19
4^th^ (Blekinge)	−4	0	4	4	5	9
5^th^ (Västerbotten)	−4	−2	3	3	4	4
Number of events avoided						
Total	91	176	252	322	386	1,228
CV death	8	15	21	26	31	99
Stroke	6	12	17	21	25	81
MI	24	46	65	83	98	315
Unstable angina	11	21	30	39	46	148
Revascularization	43	82	119	154	186	584

The estimated value of eliminating regional variations, in comparison to the highest implementing region (Halland) would be 88 QALYs, treatment of additionally 2,323 patients and avoidance of 174 CV events. These events include 14 CV deaths, 11 Strokes and 45 MIs. This equals a net value of implementation of SEK 44 million (€ 4 million). By removing the differences in regional implementation, the suboptimal implementation of icosapent ethyl would decrease by 20 · 7%. In Supplementary table 2 the expected value of specific implementation strategies is presented.

In the first scenario analysis (Table 4), the eligible population is assumed to be patients with statin treatment and indication for icosapent ethyl in RIKS-HIA at discharge, it is estimated that at least 1,986 CV events may be avoided, including 161 deaths, 131 strokes, and 510 MIs. By reaching optimal implementation an additional 22,252 patients would be treated over five years. The estimated lifetime net value of implementation is 866 QALYs or SEK 406 million (€ 35 million). In the last scenario analysis, we assume the eligible population to be patients with indication at SEPHIA FU2, about 12,107 additional patients would be treated at optimal implementation. The total number of CV events avoided is estimated to be 1,057, of which 86 deaths, 70 strokes, and 271 MIs. The results show a net value of implementation at a lifetime perspective of 426 QALYs or SEK 213 million (€ 18 million). More detailed tables of the scenario analyses results are available in Supplementary Table 3–4.

**Table 4:** Expected value of implementation estimates in scenario analyses

	RIKS-HIA	SEPHIA follow up 2
Optimal population	22,252	12,107
Current population	866	866
Difference	21,386	11,241
Value of implementation, QALYs	811	426
Value of implementation, SEK (€) (millions)	405 · 5 (35 · 0)	213 · 1 (18 · 4)
Total health gain, QALYs	5,774	3,035
Regional variations (QALYs)*		
1^st^ region	75	83
2^nd^ highest	58	23
3^rd^ highest	21	22
4^th^ highest	9	19
5^th^ highest	0 · 5	13
Total number of events avoided		
Total	1,986	1,057
CV death	161	86
Stroke	131	70
MI	510	271
Unstable angina	240	128
Revascularization	944	503

Note: *The order of regional variations differs between RIKS-HIA and SEPHIA FU2; RIKS-HIA: Halland, Västernorrland, Skåne, Västra Götaland, Östergötland. SEPHIA FU2: Halland, Blekinge, Västernorrland, Skåne, Sörmland

### Expected value of optimal implementation by region

The regional analysis for the eligible population in SEPHIA FU1 (Table 5) shows large variations in implementation between regions. Regional implementation differences account for on average 16% of the suboptimal implementation, varying from 12 · 37% (Blekinge) to 21 · 32% (Uppsala). Delayed implementations for regions implementing icosapent ethyl year two account for around 21% of the suboptimal implementation and for regions assumed to implement year three about 41%. The regional results are not totally consistent with the national ones due to the variation in the implementation timing (delayed) and some patients with unknown regional residence (*n* = 7).

**Table 5:** Expected value of optimal and improved implementation per region at first follow-up visit post MI (SEPHIA FU1)

	Optimal implementation				Improved implementation, QALYs			
	Patients	SEK*	QALYs	Events**	$$$50{\rm{\% }}$$$	$$$75{\rm{\% }}$$$	Delayed	Regional ***, Halland
Stockholm	1,898	36 · 0	72	173	34	53	-	13
Uppsala	400	7 · 6	15	37	7	11	-	3
Sörmland	447	8 · 5	17	41	8	12	-	3
Östergötland	613	11 · 6	23	59	11	17	-	4
Jönköping	545	10 · 3	21	50	10	15	-	4
Kronoberg	370	7 · 0	14	35	7	10	6	2
Kalmar	343	6 · 5	13	32	6	10	3	2
Gotland	107	2 · 0	4	10	2	3	2	1
Blekinge	272	5 · 2	10	27	5	8	2	1
Skåne	1 708	32 · 4	65	161	29	47	-	9
Halland	298	5 · 7	11	29	4	8	-	-
VGR	2 217	42 · 0	84	205	39	62	-	15
Värmland	403	7 · 6	15	39	7	11	6	2
Örebro	424	8 · 0	16	40	8	12	-	3
Västmanland	381	7 · 2	14	35	7	11	-	3
Dalarna	526	10 · 0	20	49	10	15	-	4
Gävleborg	483	9 · 2	18	44	9	14	-	4
Västernorrland	520	9 · 9	20	52	9	14	-	3
Jämtland	273	5 · 2	10	26	5	8	2	2
Västerbotten	359	6 · 8	14	35	6	10	3	2
Norrbotten	526	10 · 0	20	49	10	15	4	3

Note: *In millions. **Total decrease in number of CV events. ***Highest implementing region

To ensure an improved level of implementation up to 50% of the eligible population would result in health gains varying from 2 QALYs and SEK 1 million (Gotland) to 39 QALYs and SEK 19 · 6 million (Västra Götaland). Further an improvement to ensure a level of implementation at 75% equals a net value of improved implementation value varying from 6 to 62 QALYs or SEK 1 · 5 million to 30 · 8 million per region. Extended annual estimates and 5-point MACE per region can be found in supplementary Table 5–25.

### Sensitivity analyses

**Fig. 2:**
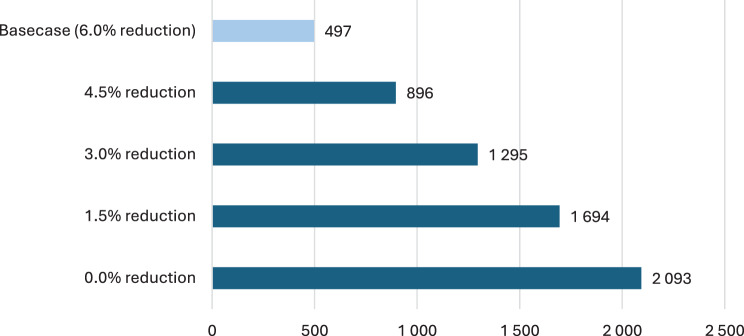
Expected value of implementation estimate (QALYs) at different levels of relative risk reduction of icosapent ethyl

Sensitivity analyses were performed by changing the assumed uncertainty of icosapent ethyl discussed by different authorities. The analyses were performed by changing the relative risk (RR) reduction of icosapent ethyl by 1 · 5% (See Fig. 2). The results show an increased net value of implementation estimate in all cases. By decreasing the reduction of the RR to 3%, which is the highest number of uncertainties assumed by NICE, FDA and EMA, the net value of implementation estimate is 1,295 QALYs, SEK 647 · 7 million and an avoidance of 1 355 CV events, including 116 CV deaths, 91 strokes and 351 MIs.

## Discussion

This study provides an upper limit of the value that could be gained by improving suboptimal implementation of icosapent ethyl in five years, by estimating the prospective net value of implementation in Sweden. The results show significant health gains in eliminating implementation variations between and across regions in Sweden. The total opportunity cost due to suboptimal implementation in clinical practice in Sweden is estimated to be about 500 QALYs and more than 1200 CV events that may be avoided. In monetary terms this is equivalent to a cost of SEK 249 million (€ 21 million), indicating that implementation strategies up to this cost are cost-effective given that they result in optimal implementation. This means that suboptimal implementation is associated with avoidable losses in both health and monetary terms. Optimal implementation of icosapent ethyl is projected to generate a total health gain of 3,538 QALYs at a total cost of SEK 1.5 billion. While this reflects the overall investment required to treat all eligible patients, the net value of implementation, i.e. defined as the difference in cost and health outcomes between optimal and current implementation, is equivalent to the previously stated opportunity costat 497 QALYs or SEK 249 million (€21 million). This net value indicates that implementation strategies costing up to SEK 249 million would be considered cost-effective, provided they succeed in achieving optimal uptake. On a per-patient basis, this corresponds to a net value of SEK 18,960 (€1,636). Icosapent ethyl has been approved and reimbursed in Sweden, with a cost-effectiveness ratio (ICER) of approximately SEK 430,000 per QALY gained, supporting its value in the Swedish healthcare context. This corresponds to treating approximately 3–4 patients to gain one QALY.

Eliminating regional variations will result in an estimated health gain equal to 88 QALYs and 174 avoided CV events in Sweden. Further investigations are needed to explain the large regional variations and why the implementations differ between regions. The significant regional disparities in icosapent ethyl implementation in Sweden raise concerns about healthcare equity. It may be of interest to explore what regions with higher implementation are doing differently, along with understanding how this can be adopted by others. For instance, at SEPHIA FU1 the average level of implementation nationwide is 6 · 2%, in contrast to the highest implementing region (Halland) where it is 22 · 8%. This indicates that patients’ access to this treatment largely depends on their place of residence and that there is uneven access to treatment that may decrease the risk of CV events. It was also noted that there are geographical clusters where implementation was delayed, particularly in Blekinge, Kronoberg, Kalmar, Gotland, as well as Jämtland, Västerbotten, and Norrbotten. This may be due to proximity to one another, leading to interconnected challenges and/or mutual influence.

A reason for suboptimal implementation could be the lack of clear guidelines for implementation of icosapent ethyl in national and regional therapy recommendations or local hospital protocols [25]. In this study, we did not specifically investigate the reasons behind the low uptake of icosapent ethyl. However, several plausible explanations can be proposed. Drawing on the domains outlined in the Consolidated Framework for Implementation Research (CFIR), we suggest that the primary barriers to the implementation of icosapent ethyl in Sweden are situated within the domain Inner setting. Factors related to Networks and Communications appear to be especially relevant. These include the absence of clear clinical guidelines, limited practitioner education, peer influence, and prevailing working conditions. Additionally, a lack of available resources is likely to constitute another significant barrier to implementation. This phenomenon is not unique to icosapent ethyl but can be generalized to the majority of novel medications within the cardiovascular therapy area in Europe. Studies show that new drugs typically follow a classic diffusion pattern, where after adoption by opinion leaders, less integrated physicians gradually adopt the drug, suggesting a potential wider acceptance of icosapent ethyl in the future [8, 13, 26–28]. Suboptimal prescribing practices can also be attributed to clinicians’ failure to keep up with new research findings as well as physicians’ risk preferences. To address these influences, tailored intervention strategies based on behavioral change and knowledge translation models are necessary. Continuous updating of knowledge in response to new evidence is essential to ensure that research findings have a meaningful impact on clinical practice and to ensure identification of eligible patients. Limited practitioner education may also cause uncertainty about the efficacy and side effects of icosapent ethyl, particularly in patients with multimorbidity already using multiple medications, where preventive treatments may be viewed as lower priority. Furthermore, initiation of treatment often requires specialist visits, which can be a barrier since many patients are primarily managed in primary care. These factors add complexity to real-world implementation. It is important to note that this study focuses on incident patients starting treatment and does not address challenges related to prevalent patients already receiving therapy. The slow uptake of icosapent ethyl reflects a wider issue in the implementation of secondary prevention therapies. Addressing these barriers may serve as a model for improving implementation of other similar cost-effective treatments, benefiting a broader patient population.

Another possible explanation for the low uptake of icosapent ethyl in Sweden is that icosapent ethyl may be prescribed primarily to patients with TG levels well above the indicated 1 · 7 mmol/l. On average, 40% of patients for whom icosapent ethyl is recommended are currently or have previously been treated with fibrates which is typically prescribed for patients with very high TG levels (TG > 10 mmol/l) to reduce the risk of hyperkalemia [29]. According to data from the Swedeheart registry, 23% of post-myocardial infarction (MI) patients in the SEPHIA FU1 cohort have TG levels between 1 · 7 and 5 · 7 mmol/L, with 75% of these falling between 1.7 and 2 · 6 mmol/L. Only 0 · 3% of the patients have TG levels exceeding 5 · 7 mmol/l. These findings suggests that many patients who meet the indication for icosapent ethyl may not be identified for treatment if clinical attention is focused on markedly elevated TG levels rather than the recommended threshold of 1 · 7 mmol/L.

Icosapent ethyl significantly reduces the risk of cardiovascular events, particularly in high-risk patients on statin therapy. However, safety concerns may affect prescribing behavior. An increased risk of atrial fibrillation has been reported in clinical trials and real-world studies, and bleeding risk may rise when combined with antithrombotic therapy [5, 30, 31]. Although such risks are generally low and the treatment is well tolerated, especially early in the course, these concerns may contribute to hesitancy among clinicians. Addressing these perceptions is important to ensure eligible patients benefit from this effective therapy. Furthermore, the potential availability and use of alternative omega-3 formulations may influence prescriber choices and patient access, and should be considered when defining and estimating optimal implementation in the Swedish context.

In this analysis, 6% reduction in relative risk due to an eventual impact of mineral oil as placebo is used which is assumed by TLV to be the highest effect. The assumed effect is higher compared to other countries’ authorities, which may underestimate the effects of icosapent ethyl in real life. Furthermore, a recent post-hoc analysis based on REDUCE-IT, showed that patients with recent acute coronary syndrome (ACS) receiving icosapent ethyl had a greater (36%) RR reduction vs placebo compared with the entire secondary prevention population (27%) which we have considered in this analysis [32]. This shows that there may be larger health gains in systematically implementing icosapent ethyl post-ACS. As the sensitivity analyses showed, the health gains would be even greater with a lower reduction of RR.

A key limitation with this study is that expected value of implementation framework is heavily influenced by assumptions about current and optimal implementation. This analysis has made several estimations based on the available information and a survey from key opinion leaders, assessing the estimated level of implementation for the coming years and extrapolation of data for regions with missing values. A key limitation is the uncertainty regarding how current implementation will actually evolve over time. In this analysis, we assumed a continuation of the current implementation trajectory. There is also uncertainty surrounding what constitutes a perfect level of implementation. To account for this, we conservatively defined perfect implementation as 80% of the eligible population receiving icosapent ethyl, acknowledging that not all patients with an indication are suitable for treatment due to contraindications or clinical considerations. Another limitation is the use of randomized controlled trial data to calculate outcomes in real life clinical settings.

This study aimed to subcategorize the expected value of perfect (optimal) implementation to estimate the absolute and relative value of eliminating suboptimal implementation of icosapent ethyl in Sweden as well as to eliminate regional differences in implementation. Clinical practice variations in introducing icosapent ethyl will result in suboptimal treatments, inefficiency, health losses, and increased costs since icosapent ethyl is deemed to be cost-effective by TLV. By combining cost-effectiveness evidence with epidemiology from the Swedeheart registry, gains, and losses with optimal and current clinical implementation have been evaluated for each region in Sweden in a five-year perspective. We have placed an upper limit on the expected value of implementation strategies for icosapent ethyl in Sweden and aim to indicate which strategies are most valuable. While this study focuses on icosapent ethyl implementation within Sweden and the results may not be generalizable to other contexts, the findings highlight a universal challenge, regional disparities in access to therapies. Many countries with decentralized healthcare systems face similar barriers, with inconsistent prescribing practices, varying uptake and where treatment access can vary based on local policies, resources, and clinician awareness. The results underscore the need for standardized guidelines and centralized support, to ensure equitable healthcare and maximize health outcomes and economic efficiency.

## Conclusion

There are considerable health and efficiency gains in ensuring improved implementation of icosapent ethyl between and across Swedish regions. Efforts to ensure optimal implementation in Sweden could prevent hundreds of cardiovascular events and thus result in significant health gains over five years. Strategies addressing the regional variations could result in substantial health gains and enhance patient outcomes. However, challenges remain in the lack of clear national or local guidelines. The results emphasize the need for tailored interventions to address factors influencing prescribing practices and provide valuable guidance for clinical decision-making and resource allocation in this regard.

## Electronic supplementary material

Below is the link to the electronic supplementary material.

Supplementary Material 1

## Data Availability

Data available upon reasonable request to the corresponding author.
