# Supplementary material for: Expected value of implementation of icosapent ethyl in Sweden
Source: BMC Health Serv Res. 2025 Sep 15;25:1199. doi: 10.1186/s12913-025-13470-6 (PMC12439414; doi:10.1186/s12913-025-13470-6)
Supplement: Supplementary file 1 — Supplementary Material 1 [file 12913_2025_13470_MOESM1_ESM.docx]

Title: Expected value of implementation of icosapent ethyl in Sweden

Authors: Eklund Michaela^1^ MSc, Levin Lars-Åke1 PhD, Bernfort Lars^1^ PhD.

^1^Unit of Healthcare Analysis, Department of Medical and Health Sciences, Linköping University, Linköping, Sweden

Corresponding author: Michaela Eklund, [michaela.eklund@liu.se](mailto:michaela.eklund@liu.se), +46(0)13-28 89 68, Centre for Medical Technology Assessment, Department of Health, Medicine and Caring Sciences, Linköpings University, Sandbäcksgatan 7, SE-581 83 Linköping, Sweden.

Additional materials

**Uptake of icosapent ethyl in European countries.**

The uptake of icosapent ethyl is calculated based on the estimated reimbursement patient populations, prevalent and incident patients with indication, in each country.

S1 table. Uptake of icosapent ethyl one year after reimbursement decision in other European countries.

| Country | Reimbursed | Uptake | Reference patient population |
| --- | --- | --- | --- |
| Sweden | 2022 | 0.09% | [1] |
| Finland | 2022 | 0.05% | [2, 3] |
| The Netherlands | 2022 | 0.14% |  |
| The United Kingdom | 2022 | 0.27% | [4] |
| Spain | 2023 | 1.00% | [5-7] |

**Expected value of specific implementation strategies at SEPHIA follow-up 1.**

In table S1 the expected value of specific implementation strategies in the basecase analysis (SEPHIA FU1) is presented. Slow implementation is the difference between the current level of implementation and the highest observed value of implementation during the study period. That is the value of reaching the highest observed level of implementation as soon as the technology is reimbursed. Low implementation is the value of increasing the level of implementation from the highest observed to Optimal implementation (100%) as soon as the technology is reimbursed.

The results suggest that 97% of the suboptimal implementation at SEPHIA FU1 is due to low implementation and about 3% due to slow implementation.

S2 table. Expected value of specific implementation strategies at SEPHIA follow-up 1 in number of QALYs.

| Year | 0 | 1 | 2 | 3 | 4 | Total |
| --- | --- | --- | --- | --- | --- | --- |
| Slow implementation | 5 | 4 | 3 | 2 | 0 | 14 |
| Low implementation | 97 | 97 | 97 | 97 | 97 | 483 |

Extended results of Expected value of implementation in Scenario analyses.

S3 table. Expected value of implementation for patients with indication at discharge RIKS-HIA.

| Year | 0 | 1 | 2 | 3 | 4 | Total |
| --- | --- | --- | --- | --- | --- | --- |
| Optimal population | 4 450 | 4 450 | 4 450 | 4 450 | 4 450 | 22 252 |
| Current population | 114 | 134 | 169 | 202 | 245 | 863 |
| Difference | 4 336 | 4 316 | 4 282 | 4 248 | 4 206 | 21 388 |
| Value of implementation, QALYs | 164 | 164 | 162 | 161 | 159 | 811 |
| Value of implementation, SEK (€) (millions) | 82.2 | 81.8 | 81.2 | 80.5 | 79.7 | 405.5 |
| Expected value of specific implementation strategies (QALYs) | | | | | | |
| Slow implementation | 5 | 4 | 3 | 2 | 0 | 14 |
| Low implementation | 159 | 159 | 159 | 159 | 159 | 797 |
| Regional differences (QALYs) | | | | | | |
| 1st region (Halland) | 12 | 10 | 14 | 17 | 21 | 75 |
| 2nd highest (Västernorrland) | 1 | 14 | 12 | 14 | 17 | 58 |
| 3rd highest (Skåne) | 3 | 2 | 4 | 5 | 6 | 3 |
| 4th highest (Västra Götaland) | 3 | 0 | 2 | 2 | 2 | 9 |
| 5th highest (Östergötland) | -3 | -3 | 2 | 2 | 3 | 0.6 |
| Total number of events avoided | | | | | | |
| Total | 148 | 284 | 408 | 521 | 625 | 1 986 |
| CV death | 12 | 24 | 33 | 42 | 50 | 161 |
| Stroke | 10 | 19 | 27 | 34 | 41 | 131 |
| MI | 39 | 74 | 105 | 134 | 159 | 510 |
| Unstable angina | 18 | 34 | 49 | 63 | 75 | 240 |
| Revascularization | 69 | 133 | 193 | 249 | 300 | 944 |

S4 table. Expected value of implementation for patients with indication at second follow-up visit post MI (SEPHIA FU2).

| Year | 0 | 1 | 2 | 3 | 4 | Total |
| --- | --- | --- | --- | --- | --- | --- |
| Optimal population | 2 421 | 2 421 | 2 421 | 2 421 | 2 421 | 12 107 |
| Current population | 114 | 134 | 169 | 202 | 245 | 863 |
| Difference | 2307 | 2287 | 2253 | 2219 | 2177 | 11 244 |
| Value of implementation, QALYs | 87 | 87 | 85 | 84 | 83 | 426 |
| Value of implementation, SEK (€) (millions) | 43.7 | 43.4 | 42.7 | 42.1 | 41.3 | 213.2 |
| Expected value of specific implementation strategies (QALYs) | | | | | | |
| Slow implementation | 5 | 4 | 3 | 2 | 0 | 14 |
| Low implementation | 83 | 83 | 83 | 83 | 83 | 413 |
| Regional differences (QALYs) | | | | | | |
| 1st region (Halland) | 14 | 11 | 16 | 19 | 23 | 83 |
| 2nd highest (Blekinge) | -4 | 1 | 7 | 9 | 10 | 23 |
| 3rd highest (Västernorrland) | -1 | 6 | 5 | 6 | 7 | 22 |
| 4th highest (Skåne) | 3 | 2 | 4 | 5 | 6 | 19 |
| 5th highest (Sörmanland) | 4 | 1 | 2 | 3 | 3 | 13 |
| Total number of events avoided | | | | | | |
| Total | 79 | 151 | 217 | 277 | 332 | 1 057 |
| CV death | 7 | 13 | 18 | 22 | 26 | 86 |
| Stroke | 5 | 10 | 14 | 18 | 22 | 70 |
| MI | 21 | 39 | 56 | 71 | 85 | 271 |
| Unstable angina | 10 | 18 | 26 | 33 | 40 | 128 |
| Revascularization | 37 | 71 | 103 | 132 | 160 | 503 |

Extended results of expected value of implementation by region.

S5 table. Expected value of implementation in Stockholm.

| Year | 0 | 1 | 2 | 3 | 4 | Total |
| --- | --- | --- | --- | --- | --- | --- |
| Optimal population | 403 | 403 | 403 | 403 | 403 | 2 014 |
| Current population | 26 | 31 | 16 | 19 | 23 | 116 |
| Difference | 377 | 372 | 387 | 383 | 379 | 1 898 |
| Value of implementation, QALYs | 14 | 14 | 15 | 15 | 14 | 72 |
| Value of implementation, SEK (€) (millions) | 7 | 7 | 7 | 7 | 7 | 36 |
| Expected value of specific implementation strategies (QALYs) | | | | | | |
| Slow implementation | -0.10 | -0.29 | 0.28 | 0.15 | 0 | 0.05 |
| Low implementation | 14 | 14 | 14 | 14 | 14 | 72 |
| Delayed implementation | 0 | 0 | 0 | 0 | 0 | 0 |
| Regional differences (QALYs) | | | | | | |
| 1^st^ (Halland) | 2 | 1 | 3 | 3 | 4 | 13 |
| Total number of events avoided | | | | | | |
| Total | 13 | 25 | 35 | 45 | 54 | 173 |
| CV death | 1 | 2 | 3 | 4 | 4 | 14 |
| Stroke | 1 | 2 | 2 | 3 | 4 | 11 |
| MI | 3 | 6 | 9 | 12 | 14 | 44 |
| Unstable angina | 2 | 3 | 4 | 5 | 7 | 21 |
| Revascularization | 6 | 12 | 17 | 22 | 26 | 82 |

S6 table. Expected value of implementation in Uppsala.

| Year | 0 | 1 | 2 | 3 | 4 | Total |
| --- | --- | --- | --- | --- | --- | --- |
| Optimal population | 82 | 82 | 82 | 82 | 82 | 408 |
| Current population | 2 | 2 | 1 | 1 | 2 | 8 |
| Difference | 80 | 80 | 80 | 80 | 80 | 400 |
| Value of implementation, QALYs | 3 | 3 | 3 | 3 | 3 | 15 |
| Value of implementation, SEK (€) (millions) | 2 | 2 | 2 | 2 | 2 | 8 |
| Expected value of specific implementation strategies (QALYs) | | | | | | |
| Slow implementation | -0 | - 0 | 0.02 | 0.01 | 0 | 0.02 |
| Low implementation | 3 | 3 | 3. | 3 | 3 | 15 |
| Delayed implementation | 0 | 0 | 0 | 0 | 0 | 0 |
| Regional differences (QALYs) | | | | | | |
| 1^st^ (Halland) | 0.5 | 0.4 | 0.6 | 0.8 | 0.9 | 3.2 |
| Total number of events avoided | | | | | | |
| Total | 3 | 5 | 8 | 10 | 12 | 37 |
| CV death | 0 | 0 | 1 | 1 | 1 | 3 |
| Stroke | 0 | 0 | 0 | 1 | 1 | 2 |
| MI | 1 | 1 | 2 | 2 | 3 | 9 |
| Unstable angina | 0 | 1 | 1 | 1 | 1 | 4 |
| Revascularization | 1 | 2 | 4 | 5 | 6 | 17 |

S7 table. Expected value of implementation in Sörmland.

| Year | 0 | 1 | 2 | 3 | 4 | Total |
| --- | --- | --- | --- | --- | --- | --- |
| Optimal population | 95 | 95 | 95 | 95 | 95 | 477 |
| Current population | 6 | 4 | 6 | 7 | 8 | 31 |
| Difference | 90 | 91 | 90 | 89 | 87 | 447 |
| Value of implementation, QALYs | 3 | 3 | 3 | 3 | 3 | 17 |
| Value of implementation, SEK (€) (millions) | 2 | 2 | 2 | 2 | 2 | 8 |
| Expected value of specific implementation strategies (QALYs) | | | | | | |
| Slow implementation | 0.11 | 0.15 | 0.10 | 0.05 | 0 | 0.41 |
| Low implementation | 3 | 3 | 3 | 3 | 3 | 17 |
| Delayed implementation | 0 | 0 | 0 | 0 | 0 | 0 |
| Regional differences (QALYs) | | | | | | |
| 1^st^ (Halland) | 0.4 | 0.4 | 0.6 | 0.7 | 0.9 | 3 |
| Total number of events avoided | | | | | | |
| Total | 3 | 6 | 8 | 11 | 13 | 41 |
| CV death | 0 | 0 | 1 | 1 | 1 | 3 |
| Stroke | 0 | 0 | 1 | 1 | 1 | 3 |
| MI | 1 | 2 | 2 | 3 | 3 | 11 |
| Unstable angina | 0 | 1 | 1 | 1 | 2 | 5 |
| Revascularization | 1 | 3 | 4 | 5 | 6 | 20 |

S8 table. Expected value of implementation in Östergötland.

| Year | 0 | 1 | 2 | 3 | 4 | Total |
| --- | --- | --- | --- | --- | --- | --- |
| Optimal population | 131 | 131 | 131 | 131 | 131 | 653 |
| Current population | 1 | 3 | 10 | 12 | 14 | 39 |
| Difference | 130 | 128 | 121 | 119 | 116 | 613 |
| Value of implementation, QALYs | 5 | 5 | 5 | 5 | 4 | 23 |
| Value of implementation, SEK (€) (millions) | 2 | 2 | 2 | 2 | 2 | 12 |
| Expected value of specific implementation strategies (QALYs) | | | | | | |
| Slow implementation | 0.5 | 0.4 | 0.2 | 0.1 | 0 | 1.2 |
| Low implementation | 4 | 4 | 4 | 4 | 4 | 22 |
| Delayed implementation | 0 | 0 | 0 | 0 | 0 | 0 |
| Regional differences (QALYs) | | | | | | |
| 1^st^ (Halland) | 0.8 | 0.7 | 0.7 | 0.9 | 1.1 | 4.2 |
| Total number of events avoided | | | | | | |
| Total | 4 | 8 | 12 | 16 | 19 | 59 |
| CV death | 0 | 1 | 1 | 1 | 1 | 5 |
| Stroke | 0 | 1 | 1 | 1 | 1 | 4 |
| MI | 1 | 2 | 3 | 4 | 5 | 15 |
| Unstable angina | 1 | 1 | 1 | 2 | 2 | 7 |
| Revascularization | 2 | 4 | 6 | 7 | 9 | 28 |

S9 table. Expected value of implementation in Jönköping.

| Year | 0 | 1 | 2 | 3 | 4 | Total |
| --- | --- | --- | --- | --- | --- | --- |
| Optimal population | 111 | 111 | 111 | 111 | 111 | 557 |
| Current population | 2 | 1 | 2 | 3 | 3 | 12 |
| Difference | 109 | 110 | 109 | 109 | 108 | 545 |
| Value of implementation, QALYs | 4 | 4 | 4 | 4 | 4 | 21 |
| Value of implementation, SEK (€) (millions) | 2 | 2 | 2 | 2 | 2 | 10 |
| Expected value of specific implementation strategies (QALYs) | | | | | | |
| Slow implementation | 0.06 | 0.07 | 0.04 | 0.02 | 0 | 0.19 |
| Low implementation | 4 | 4 | 4 | 4 | 4 | 20 |
| Delayed implementation | 0 | 0 | 0 | 0 | 0 | 0 |
| Regional differences (QALYs) | | | | | | |
| 1^st^ (Halland) | 1 | 1 | 1 | 1 | 1 | 4 |
| Total number of events avoided | | | | | | |
| Total | 4 | 7 | 10 | 13 | 16 | 50 |
| CV death | 0 | 1 | 1 | 1 | 1 | 4 |
| Stroke | 0 | 0 | 1 | 1 | 1 | 3 |
| MI | 1 | 2 | 3 | 3 | 4 | 13 |
| Unstable angina | 0 | 1 | 1 | 2 | 2 | 6 |
| Revascularization | 2 | 3 | 5 | 6 | 8 | 24 |

S10 table. Expected value of implementation in Kronoberg.

| Year | 0 | 1 | 2 | 3 | 4 | Total |
| --- | --- | --- | --- | --- | --- | --- |
| Optimal population | 75 | 75 | 75 | 75 | 75 | 377 |
| Current population | 0 | 0 | 2 | 2 | 3 | 7 |
| Difference | 75 | 75 | 73 | 73 | 73 | 370 |
| Value of implementation, QALYs | 3 | 3 | 3 | 3 | 3 | 14 |
| Value of implementation, SEK (€) (millions) | 1 | 1 | 1 | 1 | 1 | 7 |
| Expected value of specific implementation strategies (QALYs) | | | | | | |
| Slow implementation | 0 | 0 | 0.03 | 0.02 | 0 | 0.05 |
| Low implementation | 0 | 0 | 3 | 3 | 3 | 8 |
| Delayed implementation | 3 | 3 | 0 | 0 | 0 | 6 |
| Regional differences (QALYs) | | | | | | |
| 1^st^ (Halland) | 0.5 | 0.4 | 0.6 | 0.7 | 0.8 | 3.0 |
| Total number of events avoided | | | | | | |
| Total | 3 | 5 | 7 | 9 | 11 | 35 |
| CV death | 0 | 0 | 1 | 1 | 1 | 3 |
| Stroke | 0 | 0 | 0 | 1 | 1 | 2 |
| MI | 1 | 1 | 2 | 2 | 3 | 9 |
| Unstable angina | 0 | 1 | 1 | 1 | 1 | 4 |
| Revascularization | 1 | 2 | 3 | 4 | 5 | 16 |

S11 table. Expected value of implementation in Kalmar.

| Year | 0 | 1 | 2 | 3 | 4 | Total |
| --- | --- | --- | --- | --- | --- | --- |
| Optimal population | 70 | 70 | 70 | 70 | 70 | 349 |
| Current population | 0 | 1 | 1 | 1 | 2 | 5 |
| Difference | 70 | 69 | 69 | 68 | 68 | 343 |
| Value of implementation, QALYs | 3 | 3 | 3 | 3 | 3 | 13 |
| Value of implementation, SEK (€) (millions) | 1 | 1 | 1 | 1 | 1 | 7 |
| Expected value of specific implementation strategies (QALYs) | | | | | | |
| Slow implementation | 0 | 0.02 | 0.02 | 0.01 | 0 | 0.05 |
| Low implementation | 0 | 3 | 3 | 3 | 3 | 10 |
| Delayed implementation | 3 | 0 | 0 | 0 | 0 | 3 |
| Regional differences (QALYs) | | | | | | |
| 1^st^ (Halland) | 0.5 | 0.4 | 0.5 | 0.7 | 0.8 | 2.8 |
| Total number of events avoided | | | | | | |
| Total | 2 | 5 | 7 | 8 | 10 | 32 |
| CV death | 0 | 0 | 1 | 1 | 1 | 3 |
| Stroke | 0 | 0 | 0 | 1 | 1 | 2 |
| MI | 1 | 1 | 2 | 2 | 3 | 8 |
| Unstable angina | 0 | 1 | 1 | 1 | 1 | 4 |
| Revascularization | 1 | 2 | 3 | 4 | 5 | 15 |

S12 table. Expected value of implementation in Gotland.

| Year | 0 | 1 | 2 | 3 | 4 | Total |
| --- | --- | --- | --- | --- | --- | --- |
| Optimal population | 22 | 22 | 22 | 22 | 22 | 111 |
| Current population | 0 | 0 | 1 | 1 | 1 | 4 |
| Difference | 22 | 22 | 21 | 21 | 21 | 107 |
| Value of implementation, QALYs | 1 | 1 | 1 | 1 | 1 | 4 |
| Value of implementation, SEK (€) (millions) | 0.4 | 0.4 | 0.4 | 0.4 | 0.4 | 2.0 |
| Expected value of specific implementation strategies (QALYs) | | | | | | |
| Slow implementation | 0 | 0 | 0.01 | 0.01 | 0 | 0.02 |
| Low implementation | 0 | 0 | 0.8 | 0.8 | 0.8 | 2.4 |
| Delayed implementation | 0.8 | 0.8 | 0 | 0 | 0 | 1.7 |
| Regional differences (QALYs) | | | | | | |
| 1^st^ (Halland) | 0.15 | 0.13 | 0.14 | 0.18 | 0.21 | 0.81 |
| Total number of events avoided | | | | | | |
| Total | 0.8 | 1.5 | 2.1 | 2.7 | 3.2 | 10.2 |
| CV death | 0.1 | 0.1 | 0.2 | 0.2 | 0.3 | 0.8 |
| Stroke | 0.1 | 0.1 | 0.1 | 0.2 | 0.2 | 0.7 |
| MI | 0.2 | 0.4 | 0.5 | 0.7 | 0.8 | 2.6 |
| Unstable angina | 0.1 | 0.2 | 0.3 | 0.3 | 0.4 | 1.2 |
| Revascularization | 0.4 | 0.7 | 1.0 | 1.3 | 1.5 | 4.8 |

S13 table. Expected value of implementation in Blekinge.

| Year | 0 | 1 | 2 | 3 | 4 | Total |
| --- | --- | --- | --- | --- | --- | --- |
| Optimal population | 59 | 59 | 59 | 59 | 59 | 295 |
| Current population | 0 | 3 | 6 | 7 | 8 | 23 |
| Difference | 59 | 56 | 53 | 52 | 51 | 272 |
| Value of implementation, QALYs | 2 | 2 | 2 | 2 | 2 | 10 |
| Value of implementation, SEK (€) (millions) | 1 | 1 | 1 | 1 | 1 | 5 |
| Expected value of specific implementation strategies (QALYs) | | | | | | |
| Slow implementation | 0 | 0.2 | 0.1 | 0.1 | 0 | 0.4 |
| Low implementation | 0 | 1.9 | 1.9 | 1.9 | 1.9 | 7.7 |
| Delayed implementation | 2.2 | 0 | 0 | 0 | 0 | 2.2 |
| Regional differences (QALYs) | | | | | | |
| 1^st^ (Halland) | 0.4 | 0.3 | 0.3 | 0.3 | 0.4 | 1.7 |
| Total number of events avoided | | | | | | |
| Total | 2 | 4 | 6 | 7 | 9 | 27 |
| CV death | 0 | 0 | 0 | 1 | 1 | 2 |
| Stroke | 0 | 0 | 0 | 0 | 1 | 2 |
| MI | 1 | 1 | 1 | 2 | 2 | 7 |
| Unstable angina | 0 | 0 | 1 | 1 | 1 | 3 |
| Revascularization | 1 | 2 | 3 | 3 | 4 | 13 |

S14 table. Expected value of implementation in Skåne.

| Year | 0 | 1 | 2 | 3 | 4 | Total |
| --- | --- | --- | --- | --- | --- | --- |
| Optimal population | 378 | 378 | 378 | 378 | 378 | 1 891 |
| Current population | 26 | 25 | 36 | 44 | 53 | 183 |
| Difference | 352 | 353 | 342 | 335 | 326 | 1 708 |
| Value of implementation, QALYs | 13 | 13 | 13 | 13 | 12 | 65 |
| Value of implementation, SEK (€) (millions) | 7 | 7 | 6 | 6 | 6 | 32 |
| Expected value of specific implementation strategies (QALYs) | | | | | | |
| Slow implementation | 1.0 | 1.1 | 0.6 | 0.3 | 0 | 3.0 |
| Low implementation | 12 | 12 | 12 | 12 | 12 | 62 |
| Delayed implementation | 0 | 0 | 0 | 0 | 0 | 0 |
| Regional differences (QALYs) | | | | | | |
| 1^st^ (Halland) | 1 | 1 | 2 | 2 | 3 | 9 |
| Total number of events avoided | | | | | | |
| Total | 12 | 23 | 33 | 42 | 51 | 161 |
| CV death | 1 | 2 | 3 | 3 | 4 | 13 |
| Stroke | 1 | 2 | 2 | 3 | 3 | 11 |
| MI | 3 | 6 | 9 | 11 | 13 | 41 |
| Unstable angina | 1 | 3 | 4 | 5 | 6 | 19 |
| Revascularization | 6 | 11 | 16 | 20 | 24 | 77 |

S15 table. Expected value of implementation in Halland.

| Year | 0 | 1 | 2 | 3 | 4 | Total |
| --- | --- | --- | --- | --- | --- | --- |
| Optimal population | 77 | 77 | 77 | 77 | 77 | 386 |
| Current population | 14 | 12 | 17 | 21 | 25 | 88 |
| Difference | 64 | 65 | 60 | 57 | 52 | 298 |
| Value of implementation, QALYs | 2 | 2 | 2 | 2 | 2 | 11 |
| Value of implementation, SEK (€) (millions) | 1 | 1 | 1 | 1 | 1 | 6 |
| Expected value of specific implementation strategies (QALYs) | | | | | | |
| Slow implementation | 0.4 | 0.5 | 0.3 | 0.2 | 0 | 1.4 |
| Low implementation | 2 | 2 | 2 | 2 | 2 | 10 |
| Delayed implementation | 0 | 0 | 0 | 0 | 0 | 0 |
| Regional differences (QALYs) | | | | | | |
| 1^st^ (Halland) | 0 | 0 | 0 | 0 | 0 | 0 |
| Total number of events avoided | | | | | | |
| Total | 2 | 4 | 6 | 8 | 9 | 29 |
| CV death | 0 | 0 | 0 | 1 | 1 | 2 |
| Stroke | 0 | 0 | 0 | 1 | 1 | 2 |
| MI | 1 | 1 | 2 | 2 | 2 | 7 |
| Unstable angina | 0 | 1 | 1 | 1 | 1 | 4 |
| Revascularization | 1 | 2 | 3 | 4 | 4 | 14 |

S16 table. Expected value of implementation in Västra Götaland.

| Year | 0 | 1 | 2 | 3 | 4 | Total |
| --- | --- | --- | --- | --- | --- | --- |
| Optimal population | 472 | 472 | 472 | 472 | 472 | 2 362 |
| Current population | 25 | 18 | 28 | 34 | 41 | 146 |
| Difference | 447 | 454 | 444 | 439 | 432 | 2 217 |
| Value of implementation, QALYs | 17 | 17 | 17 | 17 | 16 | 84 |
| Value of implementation, SEK (€) (millions) | 8 | 9 | 8 | 8 | 8 | 42 |
| Expected value of specific implementation strategies (QALYs) | | | | | | |
| Slow implementation | 0.6 | 0.9 | 0.5 | 0.3 | 0 | 2.2 |
| Low implementation | 16 | 16 | 16 | 16 | 16 | 82 |
| Delayed implementation | 0 | 0 | 0 | 0 | 0 | 0 |
| Regional differences (QALYs) | | | | | | |
| 1^st^ (Halland) | 2 | 2 | 3 | 3 | 4 | 15 |
| Total number of events avoided | | | | | | |
| Total | 15 | 29 | 42 | 54 | 64 | 205 |
| CV death | 1 | 2 | 3 | 4 | 5 | 17 |
| Stroke | 1 | 2 | 3 | 4 | 4 | 14 |
| MI | 4 | 8 | 11 | 14 | 16 | 53 |
| Unstable angina | 2 | 4 | 5 | 6 | 8 | 25 |
| Revascularization | 7 | 14 | 20 | 26 | 31 | 97 |

S17 table. Expected value of implementation in Värmland.

| Year | 0 | 1 | 2 | 3 | 4 | Total |
| --- | --- | --- | --- | --- | --- | --- |
| Optimal population | 84 | 84 | 84 | 84 | 84 | 422 |
| Current population | 0 | 0 | 5 | 5 | 6 | 17 |
| Difference | 84 | 84 | 79 | 79 | 78 | 405 |
| Value of implementation, QALYs | 3 | 3 | 3 | 3 | 3 | 15 |
| Value of implementation, SEK (€) (millions) | 2 | 2 | 1 | 1 | 1 | 8 |
| Expected value of specific implementation strategies (QALYs) | | | | | | |
| Slow implementation | 0 | 0 | 0.04 | 0.04 | 0 | 0.08 |
| Low implementation | 0 | 0 | 3 | 3 | 3 | 9 |
| Delayed implementation | 3 | 3 | 0 | 0 | 0 | 6 |
| Regional differences (QALYs) | | | | | | |
| 1^st^ (Halland) | 0.6 | 0.5 | 0.5 | 0.7 | 0.8 | 3.0 |
| Total number of events avoided | | | | | | |
| Total | 3 | 6 | 8 | 10 | 12 | 39 |
| CV death | 0 | 0 | 1 | 1 | 1 | 3 |
| Stroke | 0 | 0 | 1 | 1 | 1 | 3 |
| MI | 1 | 1 | 2 | 3 | 3 | 10 |
| Unstable angina | 0 | 1 | 1 | 1 | 1 | 5 |
| Revascularization | 1 | 3 | 4 | 5 | 6 | 18 |

S18 table. Expected value of implementation in Örebro.

| Year | 0 | 1 | 2 | 3 | 4 | Total |
| --- | --- | --- | --- | --- | --- | --- |
| Optimal population | 88 | 88 | 88 | 88 | 88 | 442 |
| Current population | 1 | 4 | 4 | 4 | 5 | 18 |
| Difference | 87 | 85 | 85 | 84 | 83 | 424 |
| Value of implementation, QALYs | 3 | 3 | 3 | 3 | 3 | 16 |
| Value of implementation, SEK (€) (millions) | 2 | 2 | 2 | 2 | 2 | 8 |
| Expected value of specific implementation strategies (QALYs) | | | | | | |
| Slow implementation | 0.16 | 0.05 | 0.06 | 0.03 | 0 | 0.30 |
| Low implementation | 3 | 3 | 3 | 3 | 3 | 16 |
| Delayed implementation | 0 | 0 | 0 | 0 | 0 | 0 |
| Regional differences (QALYs) | | | | | | |
| 1^st^ (Halland) | 0.6 | 0.4 | 0.6 | 0.7 | 0.9 | 3.2 |
| Total number of events avoided | | | | | | |
| Total | 3 | 6 | 8 | 11 | 13 | 40 |
| CV death | 0 | 0 | 1 | 1 | 1 | 3 |
| Stroke | 0 | 0 | 1 | 1 | 1 | 3 |
| MI | 1 | 1 | 2 | 3 | 3 | 10 |
| Unstable angina | 0 | 1 | 1 | 1 | 2 | 5 |
| Revascularization | 1 | 3 | 4 | 5 | 6 | 19 |

S19 table. Expected value of implementation in Västmanland.

| Year | 0 | 1 | 2 | 3 | 4 | Total |
| --- | --- | --- | --- | --- | --- | --- |
| Optimal population | 78 | 78 | 78 | 78 | 78 | 392 |
| Current population | 2 | 3 | 2 | 2 | 3 | 12 |
| Difference | 77 | 75 | 77 | 76 | 76 | 381 |
| Value of implementation, QALYs | 3 | 3 | 3 | 3 | 3 | 14 |
| Value of implementation, SEK (€) (millions) | 1 | 1 | 1 | 1 | 1 | 7 |
| Expected value of specific implementation strategies (QALYs) | | | | | | |
| Slow implementation | 0.03 | -0.02 | 0.03 | 0.02 | 0 | 0.07 |
| Low implementation | 3 | 3 | 3 | 3 | 3 | 14 |
| Delayed implementation | 0 | 0 | 0 | 0 | 0 | 0 |
| Regional differences (QALYs) | | | | | | |
| 1^st^ (Halland) | 0.5 | 0.4 | 0.6 | 0.7 | 0.9 | 3.0 |
| Total number of events avoided | | | | | | |
| Total | 3 | 5 | 7 | 9 | 11 | 35 |
| CV death | 0 | 0 | 1 | 1 | 1 | 3 |
| Stroke | 0 | 0 | 0 | 1 | 1 | 2 |
| MI | 1 | 1 | 2 | 2 | 3 | 9 |
| Unstable angina | 0 | 1 | 1 | 1 | 1 | 4 |
| Revascularization | 1 | 2 | 3 | 4 | 5 | 17 |

S20 table. Expected value of implementation in Dalarna.

| Year | 0 | 1 | 2 | 3 | 4 | Total |
| --- | --- | --- | --- | --- | --- | --- |
| Optimal population | 108 | 108 | 108 | 108 | 108 | 541 |
| Current population | 1 | 3 | 3 | 4 | 5 | 15 |
| Difference | 107 | 106 | 105 | 104 | 104 | 526 |
| Value of implementation, QALYs | 4 | 4 | 4 | 4 | 4 | 20 |
| Value of implementation, SEK (€) (millions) | 2 | 2 | 2 | 2 | 2 | 10 |
| Expected value of specific implementation strategies (QALYs) | | | | | | |
| Slow implementation | 0.13 | 0.08 | 0.05 | 0.03 | 0 | 0.29 |
| Low implementation | 4 | 4 | 4 | 4 | 4 | 20 |
| Delayed implementation | 0 | 0 | 0 | 0 | 0 | 0 |
| Regional differences (QALYs) | | | | | | |
| 1^st^ (Halland) | 0.7 | 0.6 | 0.8 | 1.0 | 1.2 | 4.1 |
| Total number of events avoided | | | | | | |
| Total | 4 | 7 | 10 | 13 | 15 | 49 |
| CV death | 0 | 1 | 1 | 1 | 1 | 4 |
| Stroke | 0 | 0 | 1 | 1 | 1 | 3 |
| MI | 1 | 2 | 3 | 3 | 4 | 13 |
| Unstable angina | 0 | 1 | 1 | 2 | 2 | 6 |
| Revascularization | 2 | 3 | 5 | 6 | 7 | 23 |

S21 table. Expected value of implementation in Gävleborg.

| Year | 0 | 1 | 2 | 3 | 4 | Total |
| --- | --- | --- | --- | --- | --- | --- |
| Optimal population | 101 | 101 | 101 | 101 | 101 | 506 |
| Current population | 4 | 5 | 4 | 4 | 5 | 23 |
| Difference | 97 | 97 | 97 | 97 | 96 | 483 |
| Value of implementation, QALYs | 4 | 4 | 4 | 4 | 4 | 18 |
| Value of implementation, SEK (€) (millions) | 2 | 2 | 2 | 2 | 2 | 9 |
| Expected value of specific implementation strategies (QALYs) | | | | | | |
| Slow implementation | 0.04 | 0.03 | 0.06 | 0.04 | 0 | 0.17 |
| Low implementation | 4 | 4 | 4 | 4 | 4 | 18 |
| Delayed implementation | 0 | 0 | 0 | 0 | 0 | 0 |
| Regional differences (QALYs) | | | | | | |
| 1^st^ (Halland) | 0.5 | 0.4 | 0.7 | 0.9 | 1.0 | 3.5 |
| Total number of events avoided | | | | | | |
| Total | 3 | 6 | 9 | 12 | 14 | 44 |
| CV death | 0 | 1 | 1 | 1 | 1 | 4 |
| Stroke | 0 | 0 | 1 | 1 | 1 | 3 |
| MI | 1 | 2 | 2 | 3 | 4 | 11 |
| Unstable angina | 0 | 1 | 1 | 1 | 2 | 5 |
| Revascularization | 2 | 3 | 4 | 6 | 7 | 21 |

S22 table. Expected value of implementation in Västernorrland.

| Year | 0 | 1 | 2 | 3 | 4 | Total |
| --- | --- | --- | --- | --- | --- | --- |
| Optimal population | 117 | 117 | 117 | 117 | 117 | 584 |
| Current population | 4 | 13 | 13 | 15 | 19 | 64 |
| Difference | 113 | 103 | 104 | 101 | 98 | 520 |
| Value of implementation, QALYs | 4 | 4 | 4 | 4 | 4 | 20 |
| Value of implementation, SEK (€) (millions) | 2 | 2 | 2 | 2 | 2 | 10 |
| Expected value of specific implementation strategies (QALYs) | | | | | | |
| Slow implementation | 0.6 | 0.2 | 0.2 | 0.1 | 0 | 1.1 |
| Low implementation | 4 | 4 | 4 | 4 | 4 | 19 |
| Delayed implementation | 0 | 0 | 0 | 0 | 0 | 0 |
| Regional differences (QALYs) | | | | | | |
| 1^st^ (Halland) | 0.7 | 0.2 | 0.5 | 0.6 | 0.7 | 2.6 |
| Total number of events avoided | | | | | | |
| Total | 4 | 7 | 11 | 14 | 16 | 52 |
| CV death | 0 | 1 | 1 | 1 | 1 | 4 |
| Stroke | 0 | 0 | 1 | 1 | 1 | 3 |
| MI | 1 | 2 | 3 | 3 | 4 | 13 |
| Unstable angina | 0 | 1 | 1 | 2 | 2 | 6 |
| Revascularization | 2 | 3 | 5 | 6 | 8 | 25 |

S23 table. Expected value of implementation in Jämtland.

| Year | 0 | 1 | 2 | 3 | 4 | Total |
| --- | --- | --- | --- | --- | --- | --- |
| Optimal population | 56 | 56 | 56 | 56 | 56 | 281 |
| Current population | 0 | 2 | 2 | 2 | 2 | 8 |
| Difference | 56 | 54 | 55 | 54 | 54 | 273 |
| Value of implementation, QALYs | 2 | 2 | 2 | 2 | 2 | 10 |
| Value of implementation, SEK (€) (millions) | 1 | 1 | 1 | 1 | 1 | 5 |
| Expected value of specific implementation strategies (QALYs) | | | | | | |
| Slow implementation | 0 | 0.03 | 0.03 | 0.02 | 0 | 0.07 |
| Low implementation | 0 | 2 | 2 | 2 | 2 | 8 |
| Delayed implementation | 2 | 0 | 0 | 0 | 0 | 2 |
| Regional differences (QALYs) | | | | | | |
| 1^st^ (Halland) | 0.4 | 0.3 | 0.4 | 0.5 | 0.6 | 2.1 |
| Total number of events avoided | | | | | | |
| Total | 1.9 | 3.7 | 5.3 | 6.8 | 8.1 | 25.7 |
| CV death | 0.2 | 0.3 | 0.4 | 0.5 | 0.6 | 2.1 |
| Stroke | 0.1 | 0.2 | 0.4 | 0.4 | 0.5 | 1.7 |
| MI | 0.5 | 1.0 | 1.4 | 1.7 | 2.1 | 6.6 |
| Unstable angina | 0.2 | 0.4 | 0.6 | 0.8 | 1.0 | 3.1 |
| Revascularization | 0.9 | 1.7 | 2.5 | 3.2 | 3.9 | 12.2 |

S24 table. Expected value of implementation in Västerbotten.

| Year | 0 | 1 | 2 | 3 | 4 | Total |
| --- | --- | --- | --- | --- | --- | --- |
| Optimal population | 77 | 77 | 77 | 77 | 77 | 386 |
| Current population | 0 | 2 | 7 | 8 | 10 | 27 |
| Difference | 77 | 75 | 70 | 69 | 67 | 359 |
| Value of implementation, QALYs | 3 | 3 | 3 | 3 | 3 | 14 |
| Value of implementation, SEK (€) (millions) | 1 | 1 | 1 | 1 | 1 | 7 |
| Expected value of specific implementation strategies (QALYs) | | | | | | |
| Slow implementation | 0 | 0.3 | 0.1 | 0.1 | 0 | 0.5 |
| Low implementation | 0 | 3 | 3 | 3 | 3 | 10 |
| Delayed implementation | 3 | 0 | 0 | 0 | 0 | 3 |
| Regional differences (QALYs) | | | | | | |
| 1^st^ (Halland) | 0.5 | 0.4 | 0.4 | 0.5 | 0.6 | 2.3 |
| Total number of events avoided | | | | | | |
| Total | 3 | 5 | 7 | 9 | 11 | 35 |
| CV death | 0 | 0 | 1 | 1 | 1 | 3 |
| Stroke | 0 | 0 | 0 | 1 | 1 | 2 |
| MI | 1 | 1 | 2 | 2 | 3 | 9 |
| Unstable angina | 0 | 1 | 1 | 1 | 1 | 4 |
| Revascularization | 1 | 2 | 3 | 4 | 5 | 17 |

S25 table. Expected value of implementation in Norrbotten.

| Year | 0 | 1 | 2 | 3 | 4 | Total |
| --- | --- | --- | --- | --- | --- | --- |
| Optimal population | 108 | 108 | 108 | 108 | 108 | 539 |
| Current population | 0 | 0 | 3 | 4 | 5 | 13 |
| Difference | 108 | 107 | 104 | 104 | 103 | 526 |
| Value of implementation, QALYs | 4 | 4 | 4 | 4 | 4 | 20 |
| Value of implementation, SEK (€) (millions) | 2 | 2 | 2 | 2 | 2 | 10 |
| Expected value of specific implementation strategies (QALYs) | | | | | | |
| Slow implementation | 0 | 0.18 | 0.06 | 0.03 | 0 | 0.27 |
| Low implementation | 0 | 4 | 4 | 4 | 4 | 16 |
| Delayed implementation | 4 | 0 | 0 | 0 | 0 | 4 |
| Regional differences (QALYs) | | | | | | |
| 1^st^ (Halland) | 0.7 | 0.6 | 0.8 | 0.9 | 1.1 | 4.2 |
| Total number of events avoided | | | | | | |
| Total | 4 | 7 | 10 | 13 | 16 | 49 |
| CV death | 0 | 1 | 1 | 1 | 1 | 4 |
| Stroke | 0 | 0 | 1 | 1 | 1 | 3 |
| MI | 1 | 2 | 3 | 3 | 4 | 13 |
| Unstable angina | 0 | 1 | 1 | 2 | 2 | 6 |
| Revascularization | 2 | 3 | 5 | 6 | 7 | 23 |

References

1. Tandvårds- och läkemedelsverket. Underlag för beslut om subvention – Nyansökan. Vazkepa (ikosapentetyl). 2022.

2. Patel RS, Pasea L, Soran H, Downie P, Jones R, Hingorani AD, et al. Elevated plasma triglyceride concentration and risk of adverse clinical outcomes in 1.5 million people: a CALIBER linked electronic health record study. Cardiovasc Diabetol. 2022;21(1):102.

3. Lehto S. Country report Finland – June 2018. 2018.

4. National Institute for Health and Care Excellence. Technology appraisal guidance (TA805). Icosapent ethyl with statin therapy for reducing the risk of cardiovascular events in people with raised triglycerides. 2022.

5. Wilkins E WL, Wickramasinghe K, Bhatnagar P, Leal J, Luengo-Fernandez R, Burns R, Rayner M, Townsend N. European Cardiovascular Disease Statistics 2017. European Heart Network, Brussels. 2017.

6. Perez de Isla L, Saltijeral Cerezo A, Vitale G, Gonzalez Timon B, Torres Do Rego A, Alvarez-Sala Walther LA. [Prevalence of inapropriate LDL cholesterol levels in patients with coronary disease and/or type 2 diabetes]. Rev Clin Esp. 2012;212(10):475-81.

7. De Backer G, Jankowski P, Kotseva K, Mirrakhimov E, Reiner Z, Ryden L, et al. Management of dyslipidaemia in patients with coronary heart disease: Results from the ESC-EORP EUROASPIRE V survey in 27 countries. Atherosclerosis. 2019;285:135-46.
